# Biochar: Carbon Mitigation from the Ground Up

**DOI:** 10.1289/ehp.117-a70

**Published:** 2009-02

**Authors:** David J. Tenenbaum

As multibillion-dollar projects intended to sequester carbon dioxide (CO_2_) in deep geologic storage continue to seek financial support, the fertile black soils in the Amazon basin suggest a cheaper, lower-tech route toward the same destination. Scattered patches of dark, charcoal-rich soil known as *terra preta* (Portuguese for “black earth”) are the inspiration for an international effort to explore how burying biomass-derived charcoal, or “biochar,” could boost soil fertility and transfer a sizeable amount from the atmosphere into safe storage in of CO_2_ topsoil. Although burial of biochar is just beginning to be tested in long-term, field-scale trials, studies of Amazonian *terra preta* show that charcoal can lock up carbon in the soil for centuries and improve soil fertility.

Charcoal is made by heating wood or other organic material with a limited supply of oxygen (a process termed “pyrolysis”). The products of the pyrolysis process vary by the raw material used, burning time, and temperature, but in principle, volatile hydrocarbons and most of the oxygen and hydrogen in the biomass are burned or driven off, leaving carbon-enriched black solids with a structure that resists chemical and microbial degradation. Christoph Steiner, a research scientist at the University of Georgia, says the difference between charcoal and biochar lies primarily in the end use. “Charcoal is a fuel, and biochar has a nonfuel use that makes carbon sequestration feasible,” he explains. “Otherwise there is no difference between charcoal carbon and biochar carbon.”

Charcoal is traditionally made by burning wood in pits or temporary structures, but modern pyrolysis equipment greatly reduces the air pollution associated with this practice. Gases emitted from pyrolysis can be captured to generate valuable products instead of being released as smoke. Some of the by-products can be condensed into “bio-oil,” a liquid that can be upgraded to fuels including biodiesel and synthesis gas. A portion of the noncondensable fraction is burned to heat the pyrolysis chamber, and the rest can provide heat or fuel an electric generator.

Pyrolysis equipment now being developed at several public and private institutions typically operate at 350–700°C. In Golden, Colorado, Biochar Engineering Corporation is building portable $50,000 pyrolyzers that researchers will use to produce 1–2 tons of biochar per week. Company CEO Jim Fournier says the firm is planning larger units that could be trucked into position. Biomass is expensive to transport, he says, so pyrolysis units located near the source of the biomass are preferable to larger, centrally located facilities, even when the units reach commercial scale.

## Biochar Builds Better Soil

Spanish conquistador Francisco de Orellana reported seeing large cities on the Amazon River in 1541, but how had such large populations raised their food on the poor Amazonian soils? Low in organic matter and poor at retaining plant nutrients—which makes fertilization inefficient—these soils are quickly depleted by annual cropping. The answer lay in the incorporation of charcoal into soils, a custom still practiced by millions of people worldwide, according to Steiner. This practice allowed continuous cultivation of the same Amazonian fields and thereby supported the establishment of cities.

Researchers who have tested the impact of bio-char on soil fertility say that much of the benefit may derive from biochar’s vast surface area and complex pore structure, which is hospitable to the bacteria and fungi that plants need to absorb nutrients from the soil. Steiner says, “We believe that the structure of charcoal provides a secure habitat for microbiota, which is very important for crop production.” Steiner and coauthors noted in the 2003 book *Amazonian Dark Earths* that the charcoal-mediated enhancement of soil caused a 280–400% increase in plant uptake of nitrogen.

The contrast between charcoal-enriched soil and typical Amazonian soil is still obvious, says Clark Erickson, a professor of anthropology at the University of Pennsylvania. *Terra preta* stands out, he says, because the surrounding soils in general are poor, red, oxidized, and so rich in iron and aluminum that they sometimes are actually toxic to plants. Today, patches of *terra preta* are often used as gardens, he adds.

Anna Roosevelt, a professor of anthropology at the University of Illinois at Chicago, believes *terra preta* was created accidentally through the accumulation of garbage. The dark soil, she says, is full of human cultural traces such as house foundations, hearths, cemeteries, food remains, and artifacts, along with charcoal. In contrast, Erickson says he’s sure the Amazonian peoples knew exactly what they were doing when they developed this rich soil. As evidence, he says, “All humans produce and toss out garbage, but the *terra preta* phenomenon is limited to a few world regions.”

Recent studies show that, although biochar alone does not boost crop productivity, biochar plus compost or conventional fertilizers makes a big difference. In the February 2007 issue of *Plant and Soil*, Steiner, along with Cornell University soil scientist Johannes Lehmann and colleagues, demonstrated that use of biochar plus chemical amendments (nitrogen–phosphorus–potassium fertilizer and lime) on average doubled grain yield over four harvests compared with the use of fertilizer alone. In research presented at the April 2008 235th national meeting of the American Chemical Society, Mingxin Guo, an assistant professor of agriculture at Delaware State University, found that biochar plus chemical fertilizer increased growth of winter wheat and several vegetables by 25–50% compared with chemical fertilization alone.

“Depending on the sources, biochar may supply certain amounts of phosphorus and potassium to crops but will supply little nitrogen,” says Guo. “On the other hand, biochar promotes growth of beneficial microbes and helps retain phosphorus and potassium in soil, improving crop utilization efficiency of the nutrients. Nevertheless, bio-char fertilization may initially require more nitrogen from external sources since decomposition of biochar carbon will consume available nitrogen in soil.” With the decrease in phosphorus fertilization and increase in nutrient retention, biochar should have positive effects on reducing nutrient runoff losses, according to Guo, who adds, “Since biochar fertilization enhances soil aeration and beneficial microbial activity, it will also inhibit soilborne pathogens but not above-ground pests.”

However, not all biochar performs the same. The importance of biochar’s variable chemical composition was illustrated in studies by Goro Uehara, a professor of soil science at the University of Hawaii, who grew plants both with and without biochar made from macadamia nutshells. He says, “As we added more [biochar], the plants got sicker and sicker.” Uehara’s colleague, University of Hawaii extension specialist Jonathan Deenik, says that when they repeated the experiment with a more highly carbonized version of the nutshell biochar, which contained lower levels of volatile compounds, “preliminary results in a greenhouse study showed that low-volatility [biochar] supplemented with fertilizer outperformed fertilizer alone by 60%, in a statistically significant difference.” This research was presented at the October 2008 annual meeting of the Soil Science Society of America.

## Banking Carbon

Reseachers have come to realize the use of biochar also has phenomenal potential for sequestering carbon in a warming world. The soil already holds 3.3 times as much carbon as the atmosphere, according to a proposal Steiner wrote for submission by the United Nations Convention to Combat Desertification (UNCCD) to the Ad Hoc Working Group on Long-term Cooperative Action at the 1–10 December 2008 United Nations climate conference in Poznan, Poland. However, Steiner wrote, many soils have the capacity to hold probably several hundred billions of tons more.

Plants remove CO_2_ from the atmosphere through photosynthesis, then store the carbon in their tissues. CO_2_ is released back into the atmosphere after plant tissues decay or are burned or consumed, and the CO_2_ is then mineralized. If plant materials are transformed into charcoal, however, the carbon is permanently fixed in a solid form—evidence from Amazonia, where *terra preta* remains black and productive after several thousand years, suggests that biochar is highly stable. On average, half the biochar carbon is recalcitrant and would persistently remain in soil, according to Guo.

Carbon can also be stored in soil as crop residues or humus (a more stable material formed in soil from decaying organic matter). But soil chemist Jim Amonette of the Department of Energy’s Pacific Northwest National Laboratory points out that crop residues usually oxidize into CO_2_ and are released into the atmosphere within a couple of years, and the lifetime of carbon in humus is typically less than 25 years.

The calculations for potential carbon storage can be estimated downward from the amount of atmospheric carbon that photosynthesis removes from the air each year; using figures from the Intergovernmental Panel on Climate Change, Amonette estimates that number at 61.5 billion metric tons. He says the best estimates are presented in four scenarios for carbon storage calculated by the nonprofit International Biochar Initiative (IBI), a consortium of scientists and others who advocate for research/ development and commercialization of bio-char technology. The IBI’s “moderate” scenario assumed that 2.1% of the annual total photosynthesized carbon would be available for conversion to biochar containing 40% of the carbon in the original biomass, and that incorporating this charcoal in the soil would remove half a billion metric tons of carbon from the atmosphere annually. Because the heat and chemical energy released during pyrolysis could replace energy derived from fossil fuels, the IBI calculates the total benefit would be equivalent to removing about 1.2 billion metric tons of carbon from the atmosphere each year. That would offset 29% of today’s net rise in atmospheric carbon, which is estimated at 4.1 billion metric tons, according to the Energy Information Administration.

It is these large numbers—combined with the simplicity of the technology—that has attracted a broad range of supporters. At Michigan Technological University, for example, undergraduate Amanda Taylor says she is “interested in changing the world” by sequestering carbon through biochar. Under the guidance of Department of Humanities instructor Michael Moore, Taylor and fellow students established a research group to study the production and use of bio-char as well as how *terra preta* might fit into a framework of community and global sustainability. Among other projects, the students made their own biochar in a 55-gallon drum and found that positioning the drum horizontally produced the best burn.

The numbers are entirely theoretical at this point, and any effort to project the impact of biochar on the global carbon cycle is necessarily speculative, says Lehmann. “These estimates are at best probing the theoretical potential as a means of highlighting the need to fully explore any practical potential, and these potentials need to be looked at from environmental, social, and technological viewpoints. The reason we have no true prediction of the potential is because biochar has not been fully tested at the scale that it needs to be implemented at to achieve these predictions.”

Still, Steiner stresses that other large-scale carbon-storage possibilities also face uncertainties. “Forests only capture carbon as long as they grow, and the duration of sequestration depends very much on what happens afterward,” he says. “If the trees are used for toilet paper, the capture time is very short.” Soilborne charcoal, in contrast, is more stable, he says: “The risk of losing the carbon is very small—it cannot burn or be wiped out by disease, like a forest.”

As a carbon mitigation strategy, most biochar advocates believe biochar should be made only from plant waste, not from trees or plants grown on plantations. “The charcoal should not come from cutting down the rainforest and growing eucalyptus,” says Amonette.

## A Step toward Legitimacy

Biochar took a step toward legitimacy at the December Poznan conference, when the UNCCD placed it in consideration for negotiations for use as a mitigation strategy during the second Kyoto Protocol commitment period, which begins in 2013. Under the cap-and-trade strategy that forms the backbone of the Kyoto Protocol, businesses can buy certified emission reduction (CER) credits to offset their emissions of greenhouse gases. If biochar is recognized as a mitigation technology under the Kyoto Clean Deveopment Mechanism, people who implement this technology could sell CER credits. The market price of credits would depend on supply and demand; a high enough price could help promote the adoption of the biochar process. [For more information on offsets, see “Carbon Offsets: Growing Pains in a Growing Market,” p. A62 this issue.]

The possibility that the United Nations will give its stamp of approval to bio-char as a climate mitigation strategy means the ancient innovation may finally undergo large-scale testing. “The interest is growing extremely fast, but it took many years to receive the attention,” says Steiner. “Biochar for carbon sequestration does not have strong financial support compared to carbon capture and storage through geological sequestration. [However,] biochar is much more realistic for carbon capture.”

“For now,” he says, “I think the biggest hope and advantage is carbon sequestration and the ability to address sustainable land use, food and renewable energy production, and carbon sequestration in a complementary way—not a competing way.”

## Figures and Tables

**Figure:**
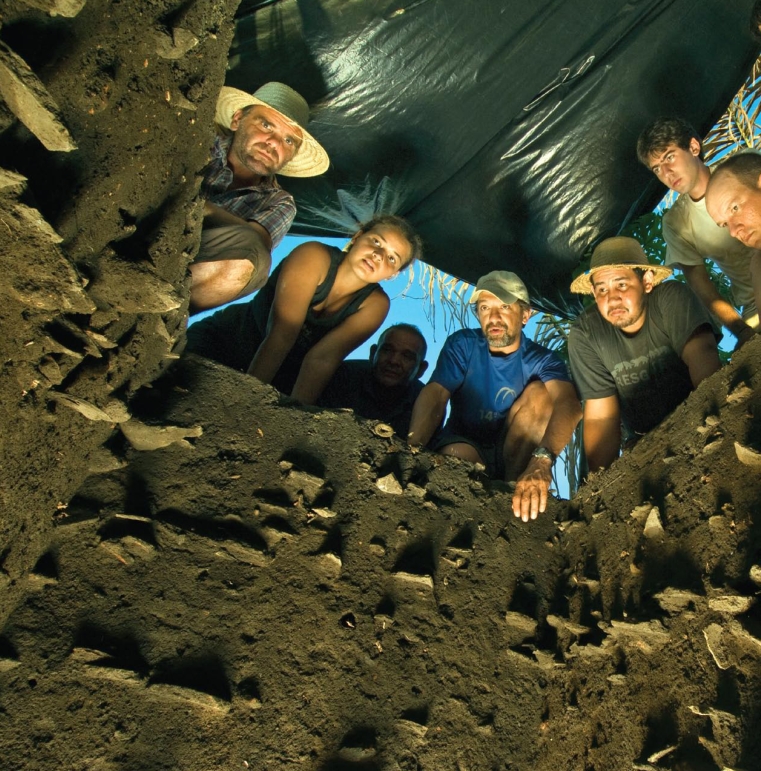
Archaeologists in Brazil peer into a pit dug into *terra preta*. The pottery shards sticking out of the walls of the pit reflect centuries’ worth of settlement—and soil amendment with biochar.

**Figure:**
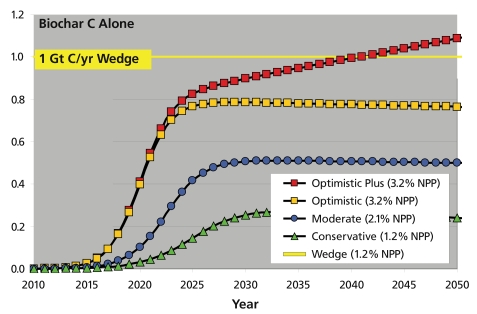
Four Scenarios for Potential Carbon Offsets

**Figure:**
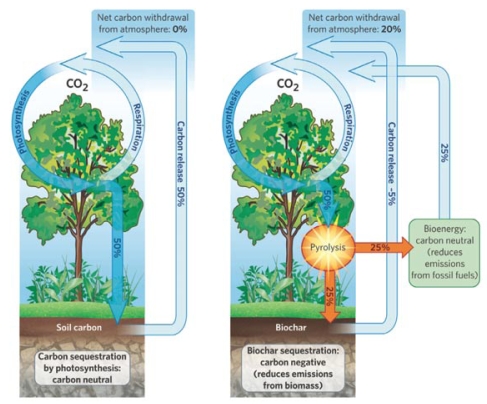
Biochar Can Be Carbon-Negative
